# Optimizing Voice Recognition Informatic Robots for Effective Communication in Outpatient Settings

**DOI:** 10.7759/cureus.44848

**Published:** 2023-09-07

**Authors:** Zuowei Meng, Hairong Liu, Alfred C Ma

**Affiliations:** 1 Nursing, Outpatient Department, Peking University Shenzhen Hospital, Shenzhen, CHN; 2 Koguan Law School, Shanghai Jiao Tong University, Shanghai, CHN; 3 Anesthesiology, Mansfield International College, Fullerton, USA

**Keywords:** patient education, healthcare trends, patient engagement, outpatient healthcare, informatic robots, voice recognition technology

## Abstract

Aim/Objective

Within the dynamic healthcare technology landscape, this research aims to explore patient inquiries within outpatient clinics, elucidating the interplay between technology and healthcare intricacies. Building upon the initial intelligent guidance robot implementation shortcomings, this investigation seeks to enhance informatic robots with voice recognition technology. The objective is to analyze users' vocal patterns, discern age-associated vocal attributes, and facilitate age differentiation through subtle vocal nuances to enhance the efficacy of human-robot communication within outpatient clinical settings.

Methods

This investigation employs a multi-faceted approach. It leverages voice recognition technology to analyze users' vocal patterns. A diverse dataset of voice samples from various age groups was collected. Acoustic features encompassing pitch, formant frequencies, spectral characteristics, and vocal tract length are extracted from the audio samples. The Mel Filterbank and Mel-Frequency Cepstral Coefficients (MFCCs) are employed for speech and audio processing tasks alongside machine learning algorithms to assess and match vocal patterns to age-related traits.

Results

The research reveals compelling outcomes. The incorporation of voice recognition technology contributes to a significant improvement in human-robot communication within outpatient clinical settings. Through accurate analysis of vocal patterns and age-related traits, informatic robots can differentiate age through nuanced verbal cues. This augmentation leads to enhanced contextual understanding and tailored responses, significantly advancing the efficiency of patient interactions with the robots.

Conclusion

Integrating voice recognition technology into informatic robots presents a noteworthy advancement in outpatient clinic settings. By enabling age differentiation through vocal nuances, this augmentation enhances the precision and relevance of responses. The study contributes to the ongoing discourse on the dynamic evolution of healthcare technology, underscoring the complex synergy between technological progression and the intricate realities within healthcare infrastructure. As healthcare continues to metamorphose, the seamless integration of voice recognition technology marks a pivotal stride in optimizing human-robot communication and elevating patient care within outpatient settings.

## Introduction

In recent years, the healthcare sector has witnessed significant advancements in technology integration, exemplified by the deployment of informatic robots across various medical settings. Notably, incorporating mobile intelligent guidance robots (IGRs) within outpatient clinics has emerged as a catalyst for enhancing operational efficiency, optimizing processes, and elevating patient experiences [1]. These autonomous robotic assistants are designed to efficiently guide patients to their designated departments, address inquiries, and contribute to the streamlined functioning of clinical environments. However, like the potential for misunderstandings among human conversational partners, informatic robots encounter distinct challenges when attempting to decode patient questions and provide precise responses. This discourse delves into the intricacies of these challenges and the complexities they face in striving to offer accurate guidance. From enigmatic queries to ambiguous inputs, this exposition unveils the multifaceted interplay of technology, language, and human interaction, emphasizing an evolutionary pathway toward achieving more refined healthcare automation.

In 2021, we published a comprehensive assessment in Cureus, critically examining the initial implementation of IGRs within an outpatient clinic setting [2]. Significantly, the core functionality of the IGR revolves around facilitating patient self-service and reducing human-to-human interactions in outpatient contexts. Critical aspects of the IGR's repertoire include intelligent navigation, triage facilitation, and educational provisions. However, the study findings underscored certain limitations, particularly in the robot's performance within challenging auditory environments. This limitation, in turn, introduced inaccuracies in the responses provided, resulting in the successful resolution of fewer than 50% of communication inquiries. In addition, the investigation also unveiled unexpected instances of misuse and abuse of the IGR. The cited article comprehensively elucidates the functions and applications of the IGR.

## Materials and methods

To address inefficiencies such as frivolous inquiries and childlike interactions, this study involves augmenting the IGR with voice recognition technology. The primary objective of this augmentation is to analyze users' vocal patterns and discern age-related vocal characteristics, thereby enabling the differentiation of age through subtle vocal nuances. This process entails utilizing advanced voice analysis techniques and machine learning algorithms [3]. This approach's foundation lies in compiling a diverse dataset comprising voice samples from individuals spanning various age cohorts. After extracting multifaceted acoustic features, including pitch, formant frequencies, spectral attributes, and vocal tract length, we implemented Mel-Frequency Cepstral Coefficients (MFCCs) to facilitate speech and audio processing, culminating in comprehensive classification analysis.

Voice recognition operates on algorithms that evaluate vocal cadences and establish correlations with age-associated vocal attributes. It's crucial to acknowledge that while voice recognition technology possesses inherent limitations that may affect absolute accuracy, it grants the capability to approximate users' ages. By leveraging the IGR's proficiency in comprehending and analyzing conversational context, the robot can glean generational references and contextual cues, thereby facilitating an estimative boundary of the user's age range.

Subsequently, in November 2022, an enhanced IGR equipped with voice recognition technology was deployed at the central lobby of an outpatient setting for 20 days, mirroring the temporal framework of the previous 2019 study. Following data accrual, the collected dataset underwent meticulous analysis, focusing on content accuracy and the frequency of successful human-robot interactions. This investigation relied on quantitative and comparative methodologies, comprehensively assessing user inquiries and data efficiencies post-upgrade. The research attempts to pinpoint areas amenable to refinement and offers strategic insights to guide future enhancements.

## Results

In this study, 101,672 outpatient visits were documented throughout the designated study duration, resulting in 30,301 human-robot interactions transpiring within 20 days. Notably, within this extensive corpus, 22,423 interactions were classified as successful interactions, a criterion defined by the robot's provision of a relevant response to the inquiry presented. Acknowledging that the IGR could not respond to languages other than Mandarin Chinese and English due to inherent programming constraints is imperative. Furthermore, notable categories such as numerically focused queries, inquiries unrelated to the hospital context, childlike interactions, and nuisance inquiries were substantially minimized or systematically omitted from the recorded dataset.

Quantitative data comparing the two distinct study periods is presented in Table 1. Set against a dynamic technological landscape and the challenges posed by the coronavirus disease 2019 (COVID-19) pandemic, discernible trends have materialized. A reduction in outpatient clinic visits has manifested, juxtaposed by a concurrent elevation in awareness regarding the use of IGR. A substantial decrease of 32.6% in outpatient visits was observed compared to the pre-pandemic year 2019. This considerable reduction was concomitant with a notable 18.7% upswing in IGR utilization for informative inquiries, thereby underscoring an amplified recognition of the pivotal role played by IGRs in mitigating human-to-human interactions.

**Table 1:** Variations of data observed between 2019 and 2022.

	2019	2022	Percent change
Total outpatient visits	150,851	101,672	-32.6%
Human robot interactions	25,526	30,301	18.7%
Successful interactions	12,543	22,423	78.8%

**Figure 1:**
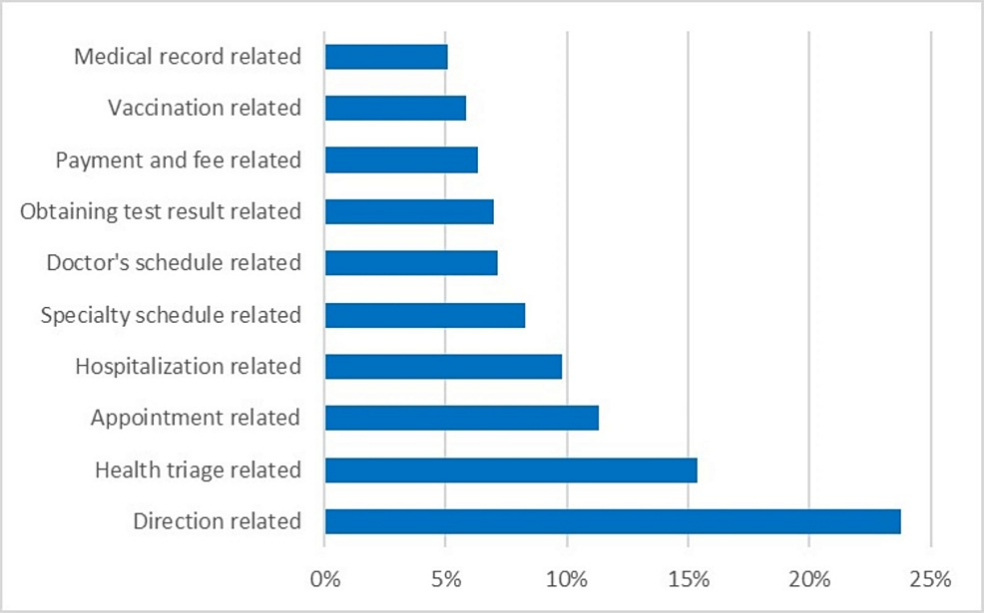
Patients’ most inquired relative subjects in 2022.

Characterized by the continuous enhancement of system applications, Figure 1 serves as a visual encapsulation of patients' inquiries, offering insight into the interplay between technology-mediated guidance and the multifaceted spectrum of patient-centric requirements. Notably, a dominant prevalence of direction-centered questions emerges as the preeminent theme. This prevalence underscores the significance of informatic robots as instrumental agents in alleviating patients' navigational concerns amidst the intricate structural layout inherent to healthcare facilities in China.

Compellingly, health triage-related inquiries and appointment issues emerge as the second and third most frequent themes within the top 10 questions. This observation carries implications that resonate with the existing healthcare framework within China. It mirrors the contemporary state of the healthcare landscape wherein the primary health system remains in a formative phase, accompanied by a noteworthy lack of coordination within the broader healthcare infrastructure. Simultaneously, appointment-related concerns underscore the challenges within the appointment scheduling process, potentially reflecting disparities in resource allocation and logistical intricacies.

Drawing a comparative lens to the antecedent year 2019, Figure 2 elucidates a substantive transformation in successful communication. The dimension of faulty inquiries, encompassing childlike inquiries and frivolous solicitations, experienced a discernible reduction. This tangible refinement can be posited as a testament to the ameliorating proficiency of informatic robots in contextual understanding, paving the way for increasingly nuanced and accurate responses.

**Figure 2:**
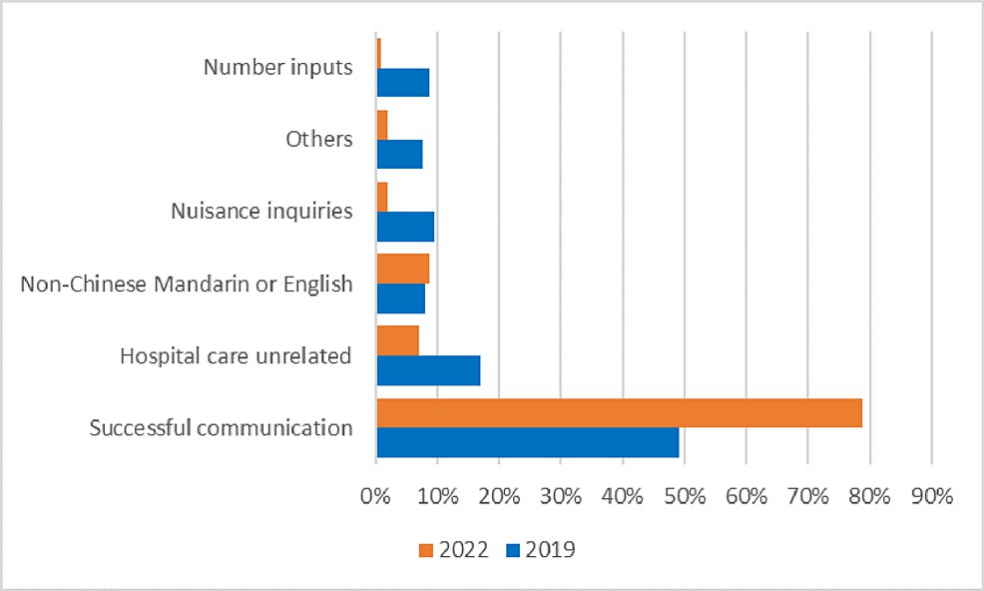
Comparative display of key parameters between 2019 and 2022.

Nevertheless, amid this trajectory of advancement, latent challenges persist. Foremost among these challenges is the constraint of language selection. In a global healthcare landscape where linguistic diversity prevails, the current scope of our informatic robots remains predominantly confined to Mandarin Chinese and English. The necessity for an expanded linguistic horizon aligns with the multifaceted nature of patient demographics and the imperative of inclusivity within healthcare automation.

## Discussion

The incorporation of voice recognition technology marks a significant milestone in the domain of informatic robots deployed in outpatient clinics. This innovative augmentation has yielded notable successes, particularly in improving patient-robot interactions. The capacity of voice recognition technology to analyze users' vocal patterns and identify age-related voice characteristics has effectively addressed a crucial communication challenge, facilitating a more tailored and efficient exchange of information between patients and robots [4]. Moreover, the benefits of voice recognition extend beyond immediate communication enhancements. As the robot processes various vocal inputs, the machine learning algorithms continually refine their understanding of vocal patterns [5]. This iterative learning process enhances the robot's future responses, contributing to an ongoing cycle of improved communication effectiveness.

Outpatient clinics consistently serve as crucial windows for patients seeking medical care. However, the introduction of informatic robots, while enhancing efficiency, potentially erodes the essential trust inherent in healthcare interactions [6]. Patients may understandably harbor reservations about entrusting their care trajectory to a mechanized entity, potentially leading to discomfort.

Mitigating these apprehensions necessitates comprehensive patient education initiatives [7]. Such initiatives enhance awareness and comprehension of informatic robots' roles and limitations. Practical measures include the provision of educational materials, both physical and digital, elucidating the robot's role in the clinic, its contributions to patient care, and the scope of inquiries it can effectively address. This understanding empowers patients to pose pertinent and informed questions [8]. Furthermore, clear guidelines for patients regarding the purpose and appropriate use of the informatic robot are essential.

By equipping patients with relevant knowledge, the significance of the interaction is reinforced, reducing the likelihood of frivolous or childlike interactions [9]. Design considerations for the informatic robot's user interface are critical, requiring an intuitive, user-friendly design that guides patients effectively through the query formulation process [10]. Clear prompts, supplemented by visual or auditory cues, facilitate a better understanding of effective interaction modalities [11]. Additionally, the interface should encourage targeted inquiries by providing suggestions or categorizing questions.

Future advancements include the integration of machine learning algorithms, which will support continuous learning from patient interactions, thereby refining responses and adapting to evolving patient needs [12]. Careful analysis of question patterns, user feedback, and transactional outcomes empowers the informatic robot to enhance its knowledge base incrementally, fine-tuning its inquiry repertoire and optimizing its ability to provide context-aware responses or seek relevant information.

Informatic robots possess the potential to catalyze a transformative shift in outpatient clinics, optimizing efficiency and elevating patient care. However, the intricacies associated with their use require careful consideration [13]. A harmonious blend of meticulous implementation, robust security measures, vigilant human oversight, and ethical guidelines is essential to harness the benefits of informatic robots while mitigating potential drawbacks [14]. Striking the right balance between technological capabilities and compassionate human touch is vital for optimal healthcare in outpatient settings.

## Conclusions

The successful integration of voice recognition technology into informatic robots has delivered substantial benefits for patient-robot interactions within outpatient clinics. This technology is a promising step toward optimizing healthcare automation by enabling more accurate age differentiation, fostering personalized engagements, and contributing to a growing knowledge base. As the healthcare landscape evolves, voice recognition technology emerges as a cornerstone in the ongoing quest to refine patient care and enrich the human-robot interaction paradigm.

In summary, the narrative surrounding the integration of informatic robots within outpatient clinics embarks on an evolutionary trajectory characterized by dynamic refinement. This narrative underscores the intricate nexus of technology and healthcare services, resonating with repercussions transcending societal and clinical paradigms. As progress is championed, the pursuit of enhancing the precision of patient inquiries resonates with overarching themes of technological amalgamation. Simultaneously, a steadfast commitment to embracing linguistic diversity epitomizes the ethos of inclusivity intrinsically linked to healthcare. Each advancement is an echelon of progress within this narrative, propelling unwavering dedication toward shaping a more nuanced and responsive healthcare future.
